# Sarracenia Purpurea in Variola

**Published:** 1864-02

**Authors:** Noah C. Levings

**Affiliations:** New York


### SARRACENIA PURPUREA IN VARIOLA.

By NOAH C. LEVINGS, M.D., of New York.

During the last month (January), I had the fortune to have under my care four children, in one family, sick with variola. Considering this to be an unusually favorable opportunity to decide upon the merits of the sarracenia as a "specific" for this disease (the children never having been vaccinated), I obtained the contused root of the sarracenia purpurea direct from Major Lane, of Halifax, the putative father of the "specific." I also requested Dr. Jacobi, of this city, to see the cases until their termination, independently of myself, and then to give me his opinion on the remedy.

The following is the history of the cases:—The first one, a boy, three years of age, unvaccinated, commenced, Tuesday morning, Jan. 12th, to complain with the usual symptoms of variola. On Thursday, the third day, the eruption appeared. We concluded to allow the disease to get under full head befor[illegible] commencing the use of the "specific." So, upon Monday af[illegible]

ternoon, the infusion of sarracenia purpurea, an ounce to the pint, was given according to published directions, that being the fifth day of the eruption, which was now distinctly pustular. The following afternoon, twenty-four hours after commencing the remedy, there was *no* increased flow of urine, no flattening nor shrivelling of the pustules, as we expected. Wednesday, the seventh day, no change in the symptoms or eruption, except a lessened fever, fuller pustules, and the central depression more positive. The eighth day, of course, the pustules began to scab, and some to break and crust. By the tenth day one-half the scabs on the face had fallen off; but on the trunk and limbs the peculiar pustules were advancing through their usual course without at all being modified by the medicine.

The second case, a boy of eight years, unvaccinated, taking the disease three days later than his brother, went through exactly the course of unmodified small-pox.

We have the same history for the third case, an infant of seven months, the eruption being preceded by convulsions, and no modification of the disease or symptoms, though administering the medicine from the commencement.

In each of these three cases the pustules went through the invariable course, being on the trunk three, and on the limbs six days later than on the face.

The fourth case, a sister of the others, ten years old, whom I vaccinated, the vaccination taking the precedent of the variola by two days, was changed to a very mild case of varioloid, having but eight or ten pustules on the face. This one was about the house each day, and had no medicine.

Presuming to know the natural course of variola, and having three cases neither modified, nor the sequence of the symptoms altered by the free use of the infusion of sarracenia purpurea, Dr. Jacobi and myself consider the sarracenia as without any medicinal virtue whatever in shortening the period of varialo, or "causing the pustules to wither or fall off" before the eighth day.

